# Dynamic Analysis and FPGA Implementation of a New Fractional-Order Hopfield Neural Network System under Electromagnetic Radiation

**DOI:** 10.3390/biomimetics8080559

**Published:** 2023-11-21

**Authors:** Fei Yu, Yue Lin, Si Xu, Wei Yao, Yumba Musoya Gracia, Shuo Cai

**Affiliations:** School of Computer and Communication Engineering, Changsha University of Science and Technology, Changsha 410114, China; 22208051680@stu.csust.edu.cn (Y.L.); xusi@stu.csust.edu.cn (S.X.); yaowei@csust.edu.cn (W.Y.); graciayumba27@gmail.com (Y.M.G.); caishuo@csust.edu.cn (S.C.)

**Keywords:** fractional-order, hopfield neural network (HNN), memristor, adomian decomposition, FPGA

## Abstract

Fractional calculus research indicates that, within the field of neural networks, fractional-order systems more accurately simulate the temporal memory effects present in the human brain. Therefore, it is worthwhile to conduct an in-depth investigation into the complex dynamics of fractional-order neural networks compared to integer-order models. In this paper, we propose a magnetically controlled, memristor-based, fractional-order chaotic system under electromagnetic radiation, utilizing the Hopfield neural network (HNN) model with four neurons as the foundation. The proposed system is solved by using the Adomain decomposition method (ADM). Then, through dynamic simulations of the internal parameters of the system, rich dynamic behaviors are found, such as chaos, quasiperiodicity, direction-controllable multi-scroll, and the emergence of analogous symmetric dynamic behaviors in the system as the radiation parameters are altered, with the order remaining constant. Finally, we implement the proposed new fractional-order HNN system on a field-programmable gate array (FPGA). The experimental results show the feasibility of the theoretical analysis.

## 1. Introduction

The nervous system comprises a vast number of neurons, which serve as the fundamental physical and functional units of neural networks [1,2,3,4,5,6]. Neurons are the fundamental units responsible for processing information in biological nervous systems. They can realize the basic functions of neurons by sensing electrical stimulation or excitation conduction [7,8,9,10,11,12]. Research has indicated that chaos phenomena can be explored within the field of neuroscience [13]. In recent years, with the advancement of research, the Hopfield neural network model has garnered significant attention in the field of neural computation [14,15,16,17]. The Hopfield neural network model and chaotic systems are typically composed of nonlinear equations and, when dealing with high-order nonlinear dynamics systems, insights and techniques can be drawn from methods in other fields [18,19]. The memristive device component possesses unique memory capabilities, serving as artificial synapses to replace native synapses within neural networks, capable of generating rich, chaotic, dynamic phenomena [20]. Bao et al. introduced a Hopfield neural network with two memristive autapse synapses per neuron, leading to the emergence of coexisting attractors in the control plane that can be shifted by toggling initial conditions [21]. Lin et al. introduced a method capable of generating n-scroll chaotic attractors by employing segmented memristors. Their approach involved investigating the electromagnetic radiation effects in a magnetized Hopfield neural network. By adjusting the control parameters of the memristors, they could arbitrarily alter the number of scrolls [22]. Yu et al. proposed a novel Hopfield neural network that introduced a new memristors instead of a synapse, resulting in highly diverse chaotic dynamics [23]. Chen et al. introduced a dual-neuron Hopfield neural network by utilizing a piecewise function in place of the activation function; complex dynamic behaviors were uncovered therein, including period-adding bifurcation, a spiking/bursting pattern, a fast-slow effect, and coexisting multi-stable patterns [24]. By manipulating the synaptic weights or activation functions of the Hopfield neural network, it is possible to generate a wide range of chaotic dynamics. Consequently, the investigation of chaotic dynamics in neural networks holds significant importance.

In modern times, individuals are exposed to various forms of radiation on a daily basis, and prolonged exposure to radiation can potentially lead to biological effects. Among the organs in the human body, the brain is considered to be the most sensitive to electromagnetic radiation (EMR) exposure [25]. Electromagnetic radiation has the potential to influence the functioning and behavior of the nervous system in the brain, and the appropriate application of electromagnetic radiation in the nervous system can be beneficial for the treatment of neurological diseases. In recent years, researchers have been employing memristors to simulate the effects of electromagnetic radiation and induced currents on neural networks. This approach facilitates the study of the interaction between electromagnetic radiation and neural systems, opening avenues for investigating the impact of radiation on neurological processes and developing novel therapeutic interventions [26,27,28], especially on learning and memory ability. Lin et al. investigated the influence of electromagnetic radiation on the chaotic dynamics of a neural network [29]. They studied a neural network model composed of three neurons and demonstrated that an increase in the amount of external electromagnetic radiation affects the dynamic behavior of the neural network by stimulating different numbers of neurons. Wan et al. introduced the concept of original memristors to describe the magnetization effects of electromagnetic radiation in a Hopfield neural network [30]. They observed the coexistence of scroll in the network and investigated the complex attractor structure variations under the influence of external pulse-controlled memristors, pulse current stimulation, and their combined effects. Ju et al. investigated the effects of electromagnetic radiation on a three-dimensional, non-autonomous Wilson neuron model based on memristors, and the study revealed various non-chaotic discharge behaviors, including different periodic, quasi-periodic, chaotic bursting, and periodic or quasi-periodic bursting patterns, arising from the influence of electromagnetic radiation [31]. Yu et al. investigated a neural network consisting of three neurons under the influence of electromagnetic radiation, leading to the observation of diverse dynamic behaviors [32]. These behaviors included coexistence of attractors, periodic patterns, discharge patterns, transient chaos, and intermittent chaos. Furthermore, the researchers implemented their findings on a hardware platform using FPGA technology.

Fractional-order systems have been a prominent research topic in the field of chaotic dynamics [33,34,35,36,37,38,39]. Fractional calculus extends the concepts of integer-order integration and differentiation to arbitrary orders, providing superior modeling capabilities for complex behaviors [35,40,41,42,43,44]. Fractional-order definitions possess superior long-term memory properties, making them particularly useful in various application domains. In fields such as medicine [45,46], finance [47], economics [48] and others, fractional-order definitions have been shown to provide more accurate descriptions of various physical phenomena compared to integer-order definitions. Al-Husban et al. introduced a two-dimensional discrete fractional-order HNN neural network, and the results indicate that, compared to integer-order counterparts, fractional-order HNN exhibits more intricate characteristics [49]. Xu et al. investigated the stability and the existence of Hopf bifurcations in neural networks with both integer-order and fractional-order neurons incorporating delays. By means of comparison, it was determined that fractional-order neural network models yield superior outcomes [50]. Xu et al. proposed a novel fractional-order HNN chaotic system and utilized this system to introduce a new image encryption algorithm based on multiple hash index chains [51]. Li et al. introduced two novel fractional-order chaotic neural networks and proposed a new image encryption algorithm based on their distinct levels of complexity [52]. Ding et al. proposed a fractional-order heterogeneous neural network model that utilizes two structurally similar, locally active memristors to couple Hindmarsh-Rose (HR) neurons and FitzHugh-Nagumo (FHN) neurons. This coupling resulted in the emergence of coexistence discharge phenomena [37]. Through a large number of studies, it has been shown that an in-depth study of the complex dynamics of fractional neural networks is worthwhile.

The rest of this article is organized as follows. In Section 2, we introduce a fractional-order HNN chaotic system based on four neurons, and its numerical solutions are computed. In Section 3, the dynamic characteristics of the proposed fractional-order system are analyzed. In Section 4, we implement the proposed novel fractional-order system on an FPGA. Section 5 concludes the paper.

## 2. Model Construction and Solution

In this section, we will primarily introduce fractional-order definitions and mathematically model the proposed fractional-order system. Subsequently, we will analyze the stability of equilibrium points in the system and solve the system using the Adomian method.

### 2.1. Fractional Calculus Definition and Its Properties

Fractional calculus has several commonly used definitions, including the Grünwald-Letnikov (G-L) definition, the Riemann-Liouville (R-L) definition, and the Caputo definition. However, these three definitions are not equivalent. The latter two definitions (R-L and Caputo) are improvements upon the Grünwald-Letnikov definition. The Riemann-Liouville definition is primarily used for studying numerical solutions of fractional partial differential equations, while the Caputo definition focuses on applications in bifurcation and chaos theory. Below are explanations of these two definitions:

**Definition** **1.** *Riemann-Liouville fractional integral definition*

(1) $$J_{t_{0}}^{q}x(t):=\frac{1}{\Gamma (q)}\int_{t_{0}}^{t}{\left(t-\tau \right)}^{q-1}x\left(\tau \right)d\tau$$

where $q\in R^{+}$ represents the q-order integral operator, Γ(·) denotes the Gamma function, and the Riemann-Liouville fractional integral satisfies the following fundamental properties:

(2) $$\left\{\begin{array}{c} J_{t_{0}}^{q}{\left(t-t_{0}\right)}^{\gamma }=\frac{\Gamma (\gamma +1)}{\Gamma (\gamma +1+q)}{\left(t-t_{0}\right)}^{\gamma +q},\\ J_{t_{0}}^{q}C=\frac{C}{\Gamma (q+1)}{\left(t-t_{0}\right)}^{q},\\ J_{t_{0}}^{q}J_{t_{0}}^{r}x\left(t\right)\left(C\right)=J_{t_{0}}^{q+r}x\left(t\right). \end{array}\right.$$

**Definition** **2.** *Caputo fractional differential definition*

(3) $${}^{*}D_{t_{0}}^{q}:=J_{t_{0}}^{m-q}D_{t_{0}}^{m}x(t)=\left\{\begin{array}{c} \frac{1}{\Gamma (m-q)}\int_{t_{0}}^{t}{\left(t-\tau \right)}^{m-q-1}x^{\left(m\right)}\left(\tau \right)d\tau ,\\ m-1<q<m,\\ \frac{d^{m}}{dt^{m}}x(t),q=m \end{array}\right.$$

where $q\in R^{+}$, m∈N, ${}^{*}D_{t_{0}}^{q}$ represents the q-order differential operator, Γ(·) denotes the Gamma function, and the Caputo fractional differential satisfies the following fundamental properties:

(4) $$\left\{\begin{array}{c} {}^{*}D_{t_{0}}^{0}=J_{t_{0}}^{0}x(t)=x(t),\\ J_{t_{0}}^{q}{(}^{*}D_{t_{0}}^{0})x(t)=x(t)-\sum_{k=0}^{m-1}x^{\left(k\right)}\left(t_{0}^{+}\right)\frac{{\left(t-t_{0}\right)}^{k}}{k!}. \end{array}\right.$$

Simply put, the ADM involves breaking down the system to be solved into three distinct components: the linear part, the nonlinear part, and the constant term. Each of these components is then individually solved, and the results are subsequently summed up. Any given fractional-order system can be represented in one of the following forms:

(5) $$\left\{\begin{array}{cc} & {}^{*}D_{t_{0}}^{q}x=Lx+Nx+g,\\ & x^{\left(k\right)}\left(t_{0}^{+}\right)=b_{k} \end{array}\right.$$

where *L*, *N*, and *g*, respectively, represent the linear, nonlinear, and constant terms of the system function. ${}^{*}D_{t_{0}}^{q}$ stands for the q-order Caputo differential operator, and $b_{k}$ represents the initial values at order *k*, where *k* = 0, …, *m* − 1, with m∈N, and m−1<q≤m. By applying the Riemann-Liouville definition and simultaneously integrating both sides of Equation (5), we arrive at the following expression:

(6) $$x=J_{t_{0}}^{q}Lx+J_{t_{0}}^{q}Nx+J_{t_{0}}^{q}g+\Phi$$

where $\Phi =\sum_{k=0}^{m-1}b_{k}\frac{{\left(t-t_{0}\right)}^{k}}{k!}$, and the solution to Equation (6) can be expressed as follows:

(7) $$x=\sum_{i=0}^{\infty }x^{i}.$$

The calculation of the nonlinear term N is a critical part of the ADM algorithm. During the solving process, the nonlinear term can be represented as:

(8) $$\left\{\begin{array}{cc} & A_{j}^{i}=\frac{1}{i!}{\left[\frac{d^{i}}{d\lambda^{i}}N\left(v_{j}^{i}\left(\lambda \right)\right)\right]}_{\lambda =0},\\ & v_{j}^{i}\left(\lambda \right)=\sum_{k=0}^{i}{\left(\lambda \right)}^{k}x_{j}^{k} \end{array}\right.$$

where *i* = 0, 1, …, *j* = 0, 1, …, *n*; *i* represents the number of terms generated in the ADM algorithm decomposition; and *j* represents the index of the variable. The form of the nonlinear term can thus be expressed as:

(9) $$Nx=\sum_{i=0}^{\infty }A^{i}\left(x^{0},x^{1},\cdots ,x^{i}\right).$$

In this manner, the solution to the equation can be expressed as:

(10) $$\begin{array}{cc} x & =\sum_{i=0}^{\infty }x^{i}\\ & =J_{t_{0}}^{q}L\sum^{\infty }x^{i}+J_{t_{0}}^{q}\sum^{\infty }A^{i}+J_{t_{0}}^{q}g+\Phi . \end{array}$$

The derivation of the formula is described as follows:

(11) $$\begin{array}{c} x^{0}=J_{t_{0}}^{q}g+\Phi ,\\ x^{1}=J_{t_{0}}^{q}Lx^{0}+J_{t_{0}}^{q}A^{0}\left(x^{0}\right),\\ \ldots ,\\ x^{i}=J_{t_{0}}^{q}Lx^{i-1}+J_{t_{0}}^{q}A^{i-1}\left(x^{0},\;x^{1},\;\ldots ,\;x^{i-1}\right),\\ \ldots \end{array}$$

The ADM algorithm has a time complexity of O(n) and a space complexity of O(1), providing significant advantages in terms of both time and space costs. This algorithm does not require a large amount of computer memory, and its solving process exhibits very high precision. Typically, only a few initial terms need to be calculated to obtain highly accurate results. For this reason, it offers significant convenience in hardware implementation. According to this algorithm, subsequent terms are numerous, but by focusing on calculating only the initial terms, the implementation complexity is greatly reduced. Therefore, it is highly suitable for FPGA implementation.

### 2.2. Fractional-Order HNN System Model

Memristors can be employed in neuroscience to simulate biological synapses and can also be utilized to describe electromagnetic induction effects [53]. Recently, a magnetic-controlled memristor has been proposed to depict the impact of electromagnetic radiation on the HNN (Hopfield neural network) [30]. The fractional-order magnetic flux memristor model chosen for this study is as follows:

(12) $$\left\{\begin{array}{c} i=W(\varphi )v,\\ D_{t_{0}}^{q}\dot{\varphi }=pv-q\varphi ,\\ W(\varphi )=a+b\varphi , \end{array}\right.$$

where *v* and *i* are the input voltage and output current, respectively; W(φ) denotes the memductance of the memristor; *a*, *b*, *p*, and *q* are the internal parameters of memristor; and φ is the magnetic flux passing through the cell membrane of neuron 2.

**Figure 1:**
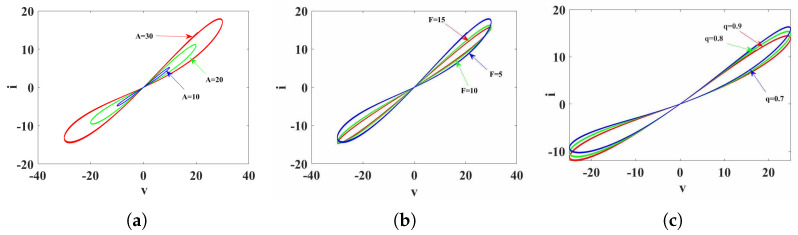
The voltage-current trajectory diagram of Equation (12). (**a**) Fixed *F* = 5, *q* = 0.9, voltage-current trajectories varying with $A_{m}$. (**b**) Fixed $A_{m}$ = 30, *q* = 0.9, voltage-current trajectories varying with *F*. (**c**) Fixed $A_{m}$ = 25, *F* = 5, voltage-current trajectories varying with *q*.

The memristor is a magnetic component with hysteresis characteristics similar to that of a magnet, and these memristors are considered to have memory functions. To investigate the dynamic properties of the memristor proposed in Equation (12), we set parameters *a* = 0.01, *b* = 0.02, *p* = 0.3, *g* = 0.8, and order *q* = 0.9 and applied an alternating voltage source $V_{m}=A_{m}sin\left(Ft\right)$. First, with a voltage frequency amplitude of *F* = 5, we varied the voltage amplitude, plotting voltage-current trajectories for different voltage amplitudes on the same plane, resulting in the voltage-current trajectory graph as depicted in Figure 1a. As $A_{m}$ remains constant, it can be observed that, with the increase in frequency *F*, the hysteresis loop area decreases. Next, with a voltage amplitude of $A_{m}$ = 30, we varied the voltage frequency, resulting in the voltage-current trajectory graph as depicted in Figure 1b. While keeping *F* constant, it is evident that, with the increase in amplitude, the hysteresis loop area increases. Finally, with $A_{m}$ = 20 and *F* = 5, voltage-current trajectory graphs were obtained for different orders, as illustrated in Figure 1c. From the voltage-current trajectory graphs depicted in Figure 1, it is evident that all hysteresis loops pass through the origin, effectively demonstrating the three key characteristics of the memristor [54].

In general, certain learning and memory behaviors in the brain can be described using feedback-type neural networks such as HNN. The HNN model possesses the ability to simulate the behavior of neurons in the human brain and serves as a powerful computational tool. It exhibits high performance in handling complex problems and provides a reliable model for simulating the dynamic behaviors of brain activity. The HNN model consisting of n neurons can be represented as follows:

(13) $$C_{i}\frac{dx_{i}}{dt}=-\frac{x_{i}}{R_{i}}+w_{ij}\sum_{j=1}^{N}tanh\left(x_{j}\right)+I_{iext},$$

where $C_{i}$, $R_{i}$ represents the capacitance and the resistance between the internal and external neurons, and *I* is the bias current at input (typically set to 0 for convenience). $x_{i}$ represents the membrane voltage on neuron *i*, and $w_{ij}$ represents the synaptic weight between neuron *i* and neuron *j*. For the voltage input to the $j^{th}$ neuron, its activation function is denoted as $tanh(x_{j})$.

**Figure 2:**
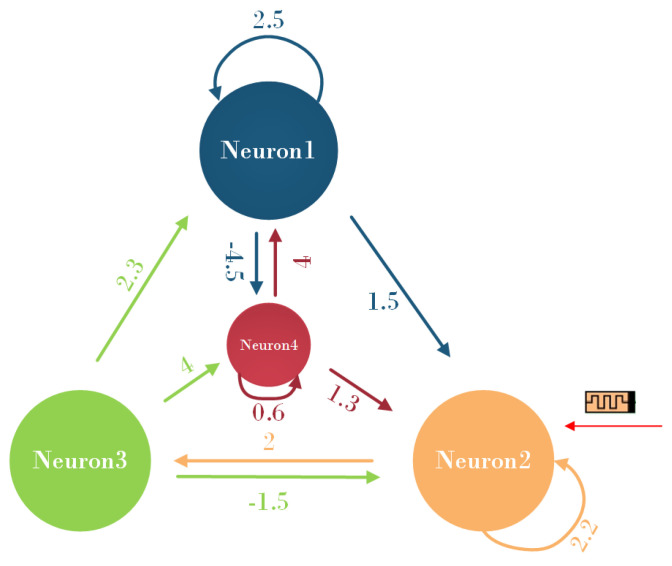
The topology of the 4-dimensional HNN model based on memristors.

Setting $R_{i}=1$, $C_{i}=1$, $I_{i}=0\;\left(i=1,2,3,4\right)$, the 4-neuron HNN model with topological connections based on these different assumptions is depicted in Figure 2.

According to the topological connectivity graph, the synaptic weight matrix is as follows:

(14) $$W=\left[\begin{array}{cccc} w_{11} & w_{12} & w_{13} & w_{14}\\ w_{21} & w_{22} & w_{23} & w_{24}\\ w_{31} & w_{32} & w_{33} & w_{34}\\ w_{41} & w_{42} & w_{43} & w_{44} \end{array}\right]=\left[\begin{array}{cccc} 2.5 & 1.5 & 0 & -4.5\\ 0 & 2.2 & 2 & 0\\ 2.3 & -1.5 & 0 & 4\\ 4 & 1.3 & 0 & 0.6 \end{array}\right].$$

Caputo differentiation is a process that involves differentiating first and then integrating. Numerical solutions based on Caputo fractional differentiation typically utilize prediction-correction methods and the Adomian decomposition method. Through analysis, it has been found that the Adomian decomposition method is more suitable for implementation on FPGA. Therefore, in this paper, the ADM is employed. Based on the Caputo definition, a novel fractional-order chaotic system under electromagnetic radiation using magnetically controlled memristors is proposed as follows:

(15) $$\left\{\begin{array}{cc} & D_{t_{0}}^{q}\dot{x}=-x+2.5\tanh(x)+1.5\tanh(y)-4.5\tanh(u)\\ & D_{t_{0}}^{q}\dot{y}=-y+2.2\tanh(y)+2\tanh(z)+ky(a+b\omega )\\ & D_{t_{0}}^{q}\dot{z}=-z+2.3\tanh(x)-1.5\tanh(y)+4\tanh(u)\\ & D_{t_{0}}^{q}\dot{u}=-u+4\tanh(x)+1.3\tanh(y)+0.6\tanh(u)\\ & D_{t_{0}}^{q}\dot{\omega }=py-g\omega \end{array},\right.$$

where *x*, *y*, *z*, and *u* are the state variables of the system, and parameter *k* represents the feedback strength of the electromagnetic radiation current. The state variable ω represents the magnetic flux on the membrane of neuron 2. *p* and *g* represent the change in magnetic flux caused by the membrane potential and the leakage of magnetic flux, respectively. *a* and *b* are two parameters associated with electromagnetic radiation.

### 2.3. Equilibrium Point Analysis

To obtain the equilibrium points, we set the left-hand side of the equation to zero and solve the equation to find all the equilibrium points of the system:

(16) $$\left\{\begin{array}{cc} & 0=-x+2.5\tanh(x)+1.5\tanh(y)-4.5\tanh(u)\\ & 0=-y+2.2\tanh(y)+2\tanh(z)+ky(a+b\omega )\\ & 0=-z+2.3\tanh(x)-1.5\tanh(y)+4\tanh(u)\\ & 0=-u+4\tanh(x)+1.3\tanh(y)+0.6\tanh(u)\\ & 0=py-g\omega \end{array}.\right.$$

From the equation above, by employing MATLAB to solve the system, the corresponding Jacobian matrix can be obtained as follows:

(17) $$\left(\begin{array}{ccccc} -1+2.5k_{1} & 1.5k_{2} & 0 & -4.5k_{4} & 0\\ 0 & -1+2.2k_{2}+k\left(a+b\omega \right) & 2k_{3} & 0 & bky\\ 2.3k_{1} & -1.5k_{2} & -1 & 4k_{4} & 0\\ 4k_{1} & 1.3k_{2} & 0 & -1+0.6k_{4} & 0\\ 0 & p & 0 & 0 & -g \end{array}\right),$$

where $k_{1}=sech^{2}\left(x\right)$, $k_{2}=sech^{2}\left(y\right)$, $k_{3}=sech^{2}\left(z\right)$, and $k_{4}=sech^{2}\left(u\right)$, where ${\mathrm{sech}}^{2}\left(i\right)=(1-\tanh^{2}\left(i\right)$ with i=x,y,z,u). Equation (16) yields $\omega =\frac{py}{g}$, with fixed parameters of *a* = 0.78, *b* = 0.1, *p* = 0.3, and *g* = 0.8, and the simplified form of Equation (16) is as follows:

(18)  z=4tanh133tanh(x)−215x+1.5tanh(y)+2.3tanh(x)−1.5tanh(y) u=133tanh(x)−215x+1.5tanh(y) H1=2.5tanh(x)−0.5u−x+1.5tanh(y) H2=2tanh(z)−0.922y+2.2tanh(y)+3800y2 H1=H2=0.

**Figure 3:**
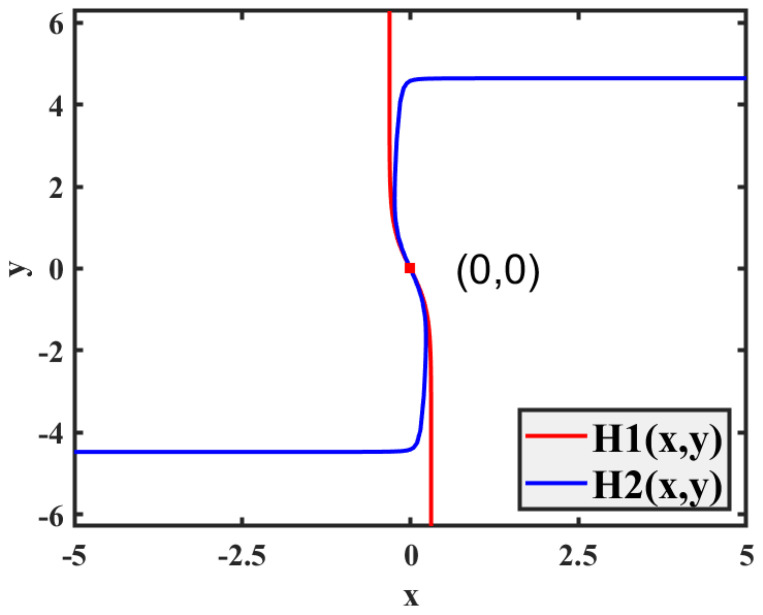
Graphical method for solving equilibrium points.

Interestingly, using a graphical method as shown in Figure 3, it can be deduced that there is only one equilibrium point, and the system consistently maintains a single equilibrium point at (0,0,0,0,0). Substituting in this equilibrium point, the characteristic function is as follows:

(19) $$\lambda^{5}-0.57\lambda^{4}+18.32\lambda^{3}-8.49\lambda^{2}+2.49\lambda +17.37.$$

The resulting eigenvalues are (−0.8,0.02914±2608i,0.65990±8720i). Unlike the stability analysis of IOD equations, the stability criterion for FOD equations at equilibrium points is:

(20) $$\left|arg(\lambda )\right|>\frac{q\pi }{2}.$$

When the above conditions are satisfied, the equilibrium point is a stable one. From this expression, it can be observed that equilibrium points of fractional-order equations are more prone to stability compared to integer-order equations.

### 2.4. Numerical Solution to the System

The solution to the system can be computed using the ADM, where the system can be decomposed into its linear and nonlinear parts, as well as a constant part:

(21) $$\begin{array}{c} \left[\begin{array}{c} L_{x}\\ L_{y}\\ L_{z}\\ L_{u}\\ L_{\omega } \end{array}\right]=\left[\begin{array}{c} -x\\ (ca-1)y\\ -z\\ -u\\ py-g\omega \end{array}\right],\\ \left[\begin{array}{c} N_{x}\\ N_{y}\\ N_{z}\\ N_{u}\\ N_{\omega } \end{array}\right]=\left[\begin{array}{c} 2.5\tanh(x)+1.5\tanh(y)-4.5\tanh(u)\\ 2.2\tanh(y)+2\tanh(z)+kb\omega y\\ 2.3\tanh(x)-1.5\tanh(y)+4\tanh(u)\\ 4\tanh(x)+1.3\tanh(y)+0.6\tanh(u)\\ 0 \end{array}\right],\\ \left[\begin{array}{c} g_{1}\\ g_{2}\\ g_{3}\\ g_{4}\\ g_{5} \end{array}\right]=\left[\begin{array}{c} 0\\ 0\\ 0\\ 0\\ 0 \end{array}\right]. \end{array}$$

For the sake of accuracy in the ADM algorithm, let the initial values of the system be set as follows:

(22)  x10=x1t0=c10 x20=x2t0=c20 x30=x3t0=c30 x40=x4t0=c40 x50=x5t0=c50.

As a result, the second term can be obtained as follows:

(23) $$\left\{\begin{array}{cc} & x_{1}^{1}=\left(-c_{1}^{0}+2.5\tanh\left(c_{1}^{0}\right)+1.5\tanh\left(c_{2}^{0}\right)-4.5\tanh\left(c_{4}^{0}\right)\right)\frac{h^{q}}{\Gamma (q+1)}\\ & x_{2}^{1}=\left(-c_{2}^{0}+2.2\tanh\left(c_{2}^{0}\right)+2\tanh\left(c_{3}^{0}\right)+{\mathrm{akc}}_{2}^{0}+bkc_{2}^{0}c_{5}^{0}\right)\frac{h^{q}}{\Gamma (q+1)}\\ & x_{3}^{1}=\left(-c_{3}^{0}+2.3\tanh\left(c_{1}^{0}\right)-1.5\tanh\left(c_{2}^{0}\right)+4\tanh\left(c_{4}^{0}\right)\right)\frac{h^{q}}{\Gamma (q+1)}\\ & x_{4}^{1}=\left(-c_{4}^{0}+4\tanh\left(c_{1}^{0}\right)+1.3\tanh\left(c_{2}^{0}\right)+0.6\tanh\left(c_{4}^{0}\right)\right)\frac{h^{q}}{\Gamma (q+1)}\\ & x_{5}^{1}=\left(pc_{2}^{0}-gc_{5}^{0}\right)\frac{h^{q}}{\Gamma (q+1)} \end{array}\right.,$$

where *h* represents the iteration step size, and Γ(·) represents the Gamma function; then, the iteration formula can be derived as follows:

(24)  c11=−c10+2.5tanhc10+1.5tanhc20−4.5tanhc40 c21=−c20+2.2tanhc20+2tanhc30+akc20+bkc20c50 c31=−c30+2.3tanhc10−1.5tanhc20+4tanhc40 c41=−c40+4tanhc10+1.3tanhc20+0.6tanhc40 c51=pc20−gc50.

By recursively applying the iteration formula, we can compute the remaining terms. However, due to the lengthy expressions for the subsequent formula, this article provides the solution for System (15) directly:

(25)  x1(t)=c10+c11hqΓ(q+1)+c12h2qΓ(2q+1)+c13h3qΓ(3q+1) x2(t)=c20+c21hqΓ(q+1)+c22h2qΓ(2q+1)+c23h3qΓ(3q+1) x3(t)=c30+c31hqΓq+1+c32h2qΓ(2q+1)+c33h3qΓ3q+1 x4(t)=c40+c41hqΓq+1)+c42h2qΓ2q+1+c43h3qΓ3q+1) x5(t)=c50+c51hqΓ(q+1)+c52h2qΓ(2q+1)+c53h3qΓ(3q+1) .

## 3. Dynamic Analysis and Numerical Simulation

In this section, we conduct a chaos dynamics analysis of the FOHNN system proposed in our study. To achieve this objective, we employ mathematical modeling and numerical simulation. We explore the system’s dynamic behavior in detail by adjusting parameters and initial conditions and by employing techniques such as computing Lyapunov exponents and bifurcation diagrams. These methods allow us to gain a comprehensive understanding of the dynamic phenomena exhibited by the system.

### 3.1. Impact of Different Orders on the Fractional-Order HNN System

**Figure 4:**
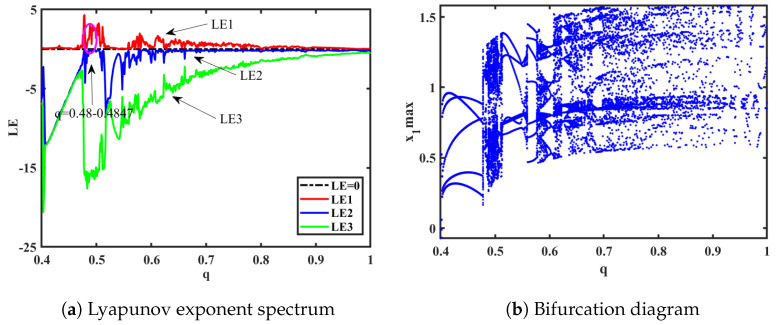
Order q∈(0.4,1).

To investigate the impact of different orders on the fractional-order HNN system, bifurcation diagrams and Lyapunov exponent spectra (considering only the first three values) were plotted and analyzed. The system parameters were set as *a* = 0.78, *b* = 0.1, *k* = 0.1, *p* = 0.3, and *g* = 0.8, with an initial value of (1, 1, −1, 0, 0) and a step size of 0.001. The Lyapunov exponent spectrum and bifurcation diagram are shown in Figure 4a and Figure 4b, respectively. From the Lyapunov exponent spectrum and bifurcation diagram, various dynamic phenomena can be observed, including the appearance of multiple periodic-to-chaotic transitions and chaotic-to-periodic transitions. The chaotic dynamics analysis of System (15) can be conducted from several aspects.

**Figure 5:**
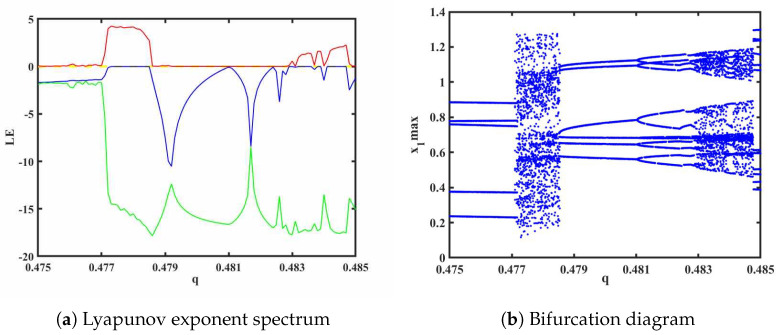
Order q∈(0.475,0.485) (The red is the value of *LE*_1_, the blue line is the value of *LE*_2_, and the green line is the value of *LE*_3_).

**Figure 6:**
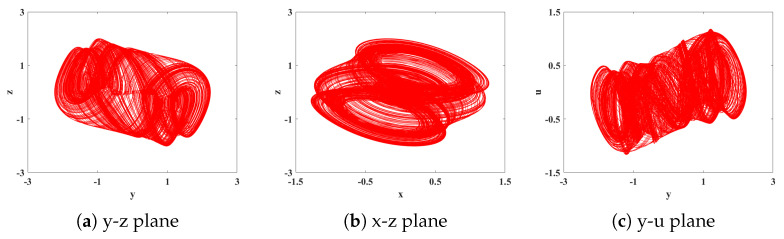
Phase diagrams of each planar chaotic attractor with order *q* = 0.478.

**Figure 7:**
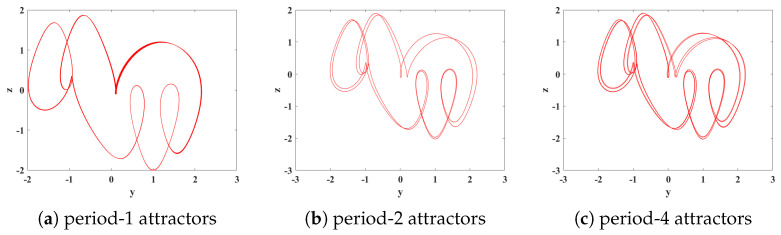
The phase portraits of different periodic attractors in the y-z plane with different values of the order *q*. (**a**) *q* = 0.481; (**b**) *q* = 0.482; (**c**) *q* = 0.4825.

**Figure 8:**
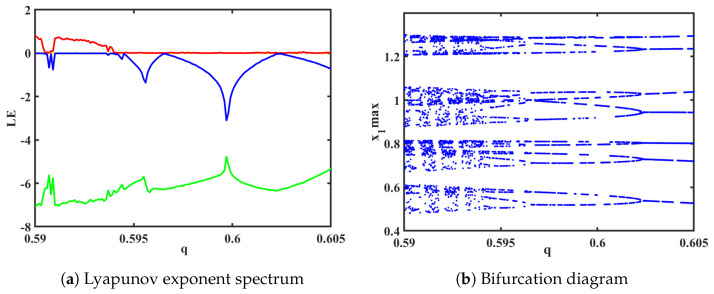
Order q∈(0.59,0.605) (The red is the value of LE1, the blue line is the value of LE2, and the green line is the value of LE3).

**Figure 9:**
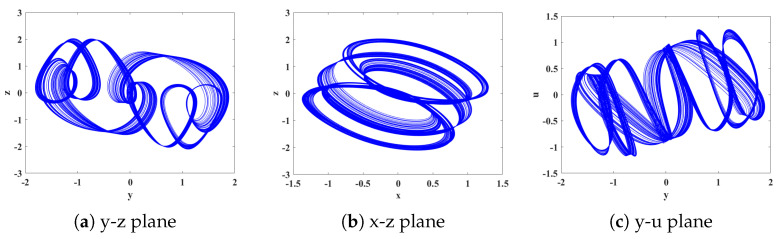
Quasi-periodic attractor phase diagram with order *q* = 0.5906.

(1) It can be seen from Figure 4 and Figure 5 that, when *q* = 0.478, $LE_{1}$ = 4.164, $LE_{2}$ = −0.006373, and $LE_{3}$ = −15.51. Due to the fact that the first Lyapunov exponent is greater than 0, the second Lyapunov exponent is close to 0 and can be considered as approximately 0, and the third Lyapunov exponent is less than 0, this indicates that the system is in a chaotic state at this moment, capable of generating chaotic attractors. The resulting attractor phase diagrams through simulations on the y-z, x-z, and y-u planes are depicted in Figure 6.

(2) When the order *q* is in the range of (0.475, 0.485), a phenomenon known as forward period-doubling bifurcation (FPDB) occurs. At *q* = 0.481, the system undergoes a bifurcation from a period-1 to a period-2, followed by further bifurcations to period-4 as the order changes, eventually leading to a chaotic state. The attractor trajectory of System (15) can be numerically simulated using MATLAB software, and the simulation results are shown in Figure 7.

(3) Based on the spectra of Lyapunov exponents and bifurcation diagrams shown in Figure 8a,b, the system transiently enters a quasi-periodic state, when order *q* is in the range (0.5905, 0.5909). The corresponding phase portraits for each plane are illustrated in Figure 9. Additionally, when order *q* is in the range (0.59, 0.605), a phenomenon of reverse period-doubling bifurcation (RPDB) occurs. When *q* = 0.594, the system transitions from a chaotic state to period-4. When *q* = 0.596, the system undergoes a reverse bifurcation from period-4 to period-2. Lastly, when *q* = 0.602, the system exhibits a reverse bifurcation to period-1.

### 3.2. Multi-Scroll Attractor with Controllable Orientation

**Figure 10:**
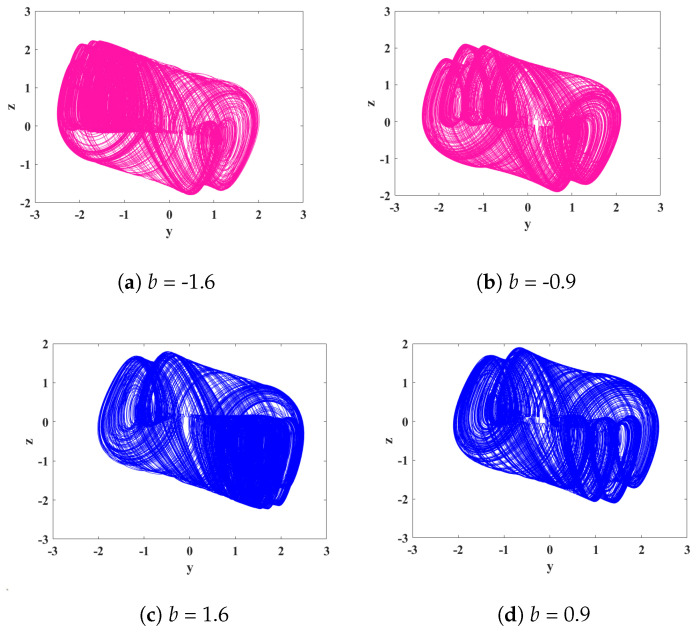
The phase portraits of multi-scroll attractors in the y-z plane for different values of parameter *b*.

During the experiment, it was discovered that, by setting the parameters as *a* = 0.78, *k* = 0.1, *p* = 2.6, and *g* = 2.5 and the order *q* = 0.478, System (15) exhibits directionally controllable multi-scroll attractors within the range of *b* parameter values (−1.6, −0.9) and (1.6, 0.9). This phenomenon is quite remarkable and has been rarely observed in other publications. Within the range of (−1.6, −0.9), the system displays multi-scroll attractors that converge towards one end, while within [1.6, 0.9], the system exhibits multi-scroll attractors that converge towards the other end. The phase portraits corresponding to this unique phenomenon are depicted in Figure 10.

### 3.3. Analysis of the Dynamic Behavior of Parameter *b* in the System

**Figure 11:**
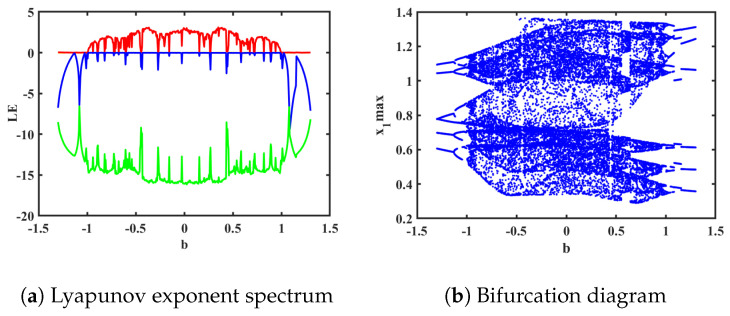
Order *q* = 0.5 (The red is the value of LE1, the blue line is the value of LE2, and the green line is the value of LE3).

**Figure 12:**
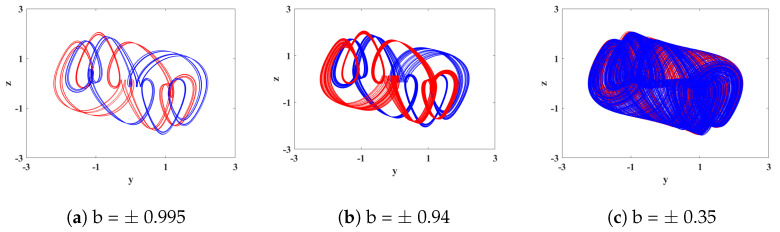
Attractor phase diagrams with different values of *b* when order *q* is 0.5. (The red color is positive, and the blue is negative).

To analyze the dynamic behavior of System (15) and study the spectra of Lyapunov exponents and bifurcation diagrams, we set the parameters as *a* = 0.78, *k* = 0.1, *p* = 0.3, *g* = 0.8, and *q* = 0.5 and the initial conditions as (1, 1, −1, 0, 0). The variation in Lyapunov exponent spectra and bifurcation diagrams for the electromagnetic parameter *b* in the range of (−1.3, 1.3) is illustrated in Figure 11. Interestingly, by observing the two graphs within the range of *b* from (−1.3, 1.3), we can observe similar symmetric dynamic phenomena. Specifically, for opposite values of *b*, similar dynamic phenomena occur at different positions. Taking *b* = 0 as the axis, the system exhibits periodic behavior within the intervals of *b* ranging from (−1.3, −0.995) to (0.995, 1.3). Moreover, within the range of *b* from (−0.995, 0) to (0, 0.995), the system demonstrates periodic, quasi-periodic, and chaotic behavior, maintaining a rough symmetric consistency. The corresponding phase portraits of the attractors are shown in Figure 12.

## 4. FPGA Hardware Implementation

FPGA has the characteristics of digitization, reconfigurability, and high flexibility, so using FPGA to implement chaotic systems is more flexible [55,56,57,58]. The FPGA hardware implementation of fractional-order neural networks is an intriguing research area in the field of neural network engineering. In this study, the development tool utilized for FPGA implementation is Vivado 2018.3. The FPGA development board used is AX7020, manufactured by ALINX, and it is equipped with the XC7Z020-2CLG400I chip produced by XILINX. The main chip operates at a frequency of 766 MHz and is powered by ARM’s Cortex-A9 core. It includes image processing acceleration modules, internal and external memory, external memory interfaces, and peripherals such as Ethernet, SD/SDIO, and UART interfaces. The development board features two 40-pin 2.54 mm dual-row connectors, of which only one row is required in this study to output the chaotic signal to an oscilloscope.

For the fractional-order HNN system, we discretize the solutions obtained from the ADM using the high-precision fourth-order Runge-Kutta (RK) method. From the solutions obtained through Adomian decomposition, it is evident that the resulting difference equations after decomposition involve a significant number of hyperbolic tangent function operations. However, from the perspective of hardware implementation and efficiency, this incurs substantial overhead and low efficiency. Implementing the solutions based on the first three terms of the decomposition is not feasible due to the large number of terms and the insufficient chip resources required for the computations. To optimize accuracy and efficiency, we choose to implement the first two terms, $x_{0}$ and $x_{1}$. However, when considering only the first two terms, $x_{0}$ and $x_{1}$, with an unchanged step size, it becomes apparent that this approach is impractical due to the significant errors it incurs. It is crucial to note that maintaining a truncation error within the range of $\pm 1\times 10^{-4}$ necessitates a reduction in the step size. Through extensive numerical computations conducted in MATLAB for the system, it was established that, by reducing the step size to $\pm 1\times 10^{-6}$ and executing $\pm 1\times 10^{6}$ iterations in Section 3.1, while maintaining an order of *q* = 0.478 and utilizing parameter values *a* = 0.78, *b* = 0.1, *k* = 0.1, *p* = 0.3, and *g* = 0.8, the maximum absolute value of $x_{2}$ is found to be $\left(3\times 10^{-5},1\times 10^{-5},3\times 10^{-5},3\times 10^{-5},1\times 10^{-6}\right)$, thereby satisfying the prescribed accuracy criteria. Likewise, in Section 3.2, with the step size set to $1\times 10^{-6}$ and the execution of $1\times 10^{6}$ iterations, while maintaining an order of *q* = 0.478 and utilizing parameter values *a* = 0.78, *b* = ±1.6, *k* = 0.1, *p* = 2.6, and *g* = 2.5, the maximum absolute value of $x_{2}$ is determined to be $\left(3\times 10^{-5},1\times 10^{-5},3\times 10^{-5},3\times 10^{-5},1\times 10^{-5}\right)$, thus satisfying the precision requirements as well. These findings underscore the importance of carefully selecting step sizes and conducting sufficient iterations to ensure the accuracy and reliability of our numerical simulations.

**Figure 13:**
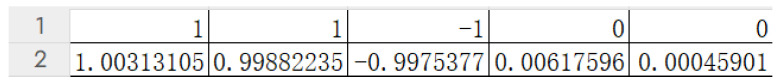
Numerical solution once of system iteration in Matlab.

**Figure 14:**
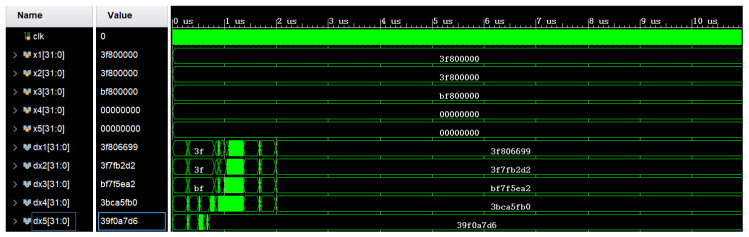
Numerical solution once of system iteration in Vivado.

The numerical simulation comparison method was employed to validate the system. Figure 13 and Figure 14, respectively, depict the numerical comparisons between MATLAB and the FPGA platform after one iteration based on the initial values (1,1,−1,0,0). Converting the hexadecimal values from dx1 to dx5 on the FPGA to decimal values yields the following results: (1.0031310,0.9988223,−0.9975377,0.0061760,0.0045901) (with the numerical results from the first iteration as an example). Based on the above verification, it is demonstrated that the system proposed in this paper can be implemented on an FPGA platform.

**Figure 15:**
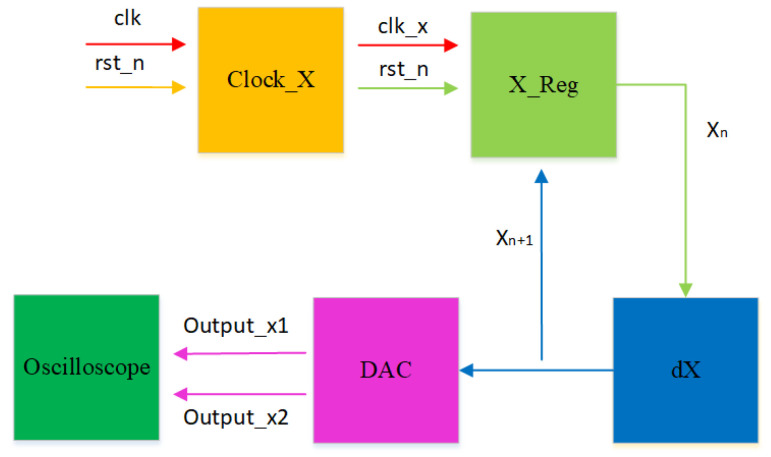
Schematic diagram of the FOHNN hardware implementation.

**Figure 16:**
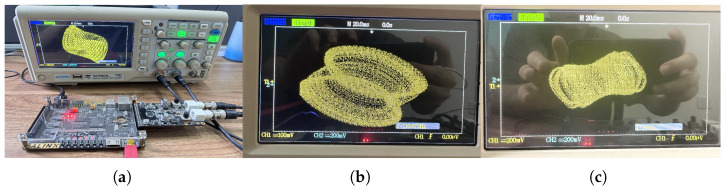
Experimental results of the FOHNN hardware implementation: (**a**) phase diagrams in the y-z plane; (**b**) phase diagrams in the x-z plane; (**c**) phase diagrams in the y-u plane.

**Figure 17:**
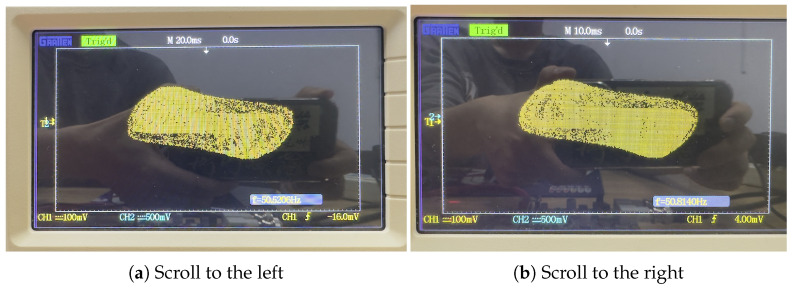
Results of directionally controllable multi-scroll hardware experiments.

The implementation process is illustrated in Figure 15. In the clk_x clock module, we set the clock period to 108 × 20 ns. With a stable output clock signal, the original computational module at the X_reg register module is updated with the results after each iteration of the initial values. This enables loop iteration to generate a stable chaotic signal. The generated digital signal is then converted to an analog signal using a digital-to-analog converter (DAC) and sent to an oscilloscope. Following the above steps, synthesis and implementation are performed using the Vivado 2018.3 simulation platform. This generates a bitstream file that is burned into the chip within the development board. Connecting the board to an oscilloscope allows for the successful generation of the corresponding attractor phase diagram. Figure 16 and Figure 17 are present the circuit implementation results of the fractional-order chaotic system’s phase portrait from Figure 6 and Figure 10, respectively. Experimental results demonstrate that the attractor phase portrait obtained from FPGA simulation is consistent with the results obtained from MATLAB simulation. Table 1 represents the resource utilization rate required for the implementation of the system project. Based on Table 1, it can be observed that the implementation of the system incurs significant resource overhead.

## 5. Conclusions

This study investigates a novel fractional-order Hopfield neural network based on magnetically controlled memristors and analyzes its dynamic characteristics under electromagnetic radiation. In this research, the complex dynamic phenomena in the neural network are induced through adjustments such as modifying synaptic weights, activation functions, and external currents. These modifications lead to the emergence of intricate chaotic behaviors within the network. Additionally, the incorporation of magnetically controlled memristors further amplifies the system’s complexity. The ADM is employed to solve the system, enabling an in-depth analysis of the influence of the order *q* and radiation parameter *b* on the system’s dynamic properties. Experimental results indicate that the system exhibits more pronounced chaotic behavior in low-order states, which is closely related to the chaotic behavior observed in neural networks. This form of chaos may have significant implications for functions such as information processing and storage within neural networks. Furthermore, we have designed and experimentally validated the proposed system through FPGA hardware implementation using the Vivado 2018.3 simulation platform, confirming the correctness of our theoretical framework. The dynamics of fractional-order neural networks continue to pose fascinating questions, including hidden transient chaos and hyperchaos phenomena. Therefore, the rich nonlinear phenomena and dynamic behaviors exhibited by the FOHNN system warrant further exploration and study. This research contributes to the understanding of fractional-order neural networks and their potential applications in complex dynamic systems, offering insights into the intricate interplay among memristors, electromagnetic radiation, and neural network dynamics. As we delve deeper into this field, we anticipate uncovering even more captivating phenomena and practical implications.

## Figures and Tables

**Table 1:** Resource utilization of FPGA chip.

Resource	Utilization	Available	Utilization %
LUT	36,824	53,200	69.22
LUTRAM	1752	17,400	10.07
FF	48,170	106,400	45.27
DSP	176	220	80.00
IO	34	125	27.20
BUFG	1	32	3.13

## Data Availability

All the data were computed using our algorithms.
